# 

**Published:** 1850-03

**Authors:** 


### CONTENTS

OF THE

### MEDICAL EXAMINER.

NEW SERIES—NO. LXIII.—MARCH, 1850.

### ORIGINAL COMMUNICATIONS.

Case of Tubercular Meningitis, occurring at St. Joseph's Hospital. Service of Dr. Stillé, - - - - - - - - - - 130

Case of Dislocation of the Crystalline Lens from a blow. By Francis West, M. D., Philadelphia, - - - - - - - - - 141

Report of the Committee appointed to examine into the condition of the Mucous Membrane of the Intestinal Canal in persons dying of Cholera, - - - - - - - - - - - 143

Wound of the Intestines, successfully treated. By Dandridge B. Hilliard, M. D., of Halifax Co., North Carolina, - - - - - 146

Philadelphia County Medical Society.—Upon the causes of the greater Mortality of Male Children, and the influence operating to change the relative proportion of the sexes at birth. By G. Emerson, M. D., - - - - - - - - - - 147

### BIBLIOGRAPHICAL NOTICES.

A Theoretical and Practical Treatise on Midwifery, including the Diseases of Pregnancy and Parturition. By P. Cazeaux, Adjunct Professor in the Faculty of Medicine of Paris, etc. etc., (adopted by the Royal Council of Public Instruction.) Translated from the second French edition, with occasional notes and a copious Index, by Robert P. Thomas, M. D., Member of the Philadelphia County Medical Society, late Demonstrator of Anatomy in the Franklin Medical College, etc., - - - - - - 154

A Treatise on Dislocation of the Shoulder. By George O. Jarvis, M. D., Author of Lectures on Fractures and Dislocations, &c., &c.; together with important cases illustrating the benefits of the Adjuster. Compiled by George Kellogg, A. M., - - 158

Transactions of the American Medical Association, - - - 161

Three Lectures Preliminary to a Course on the Principles and Practice of Surgery. Delivered on the 4th, 8th and 9th of Oct., 1849, before the Medical Class of the University of Pennsylvania. By William Gibson, M. D., LL. D., Professor of Surgery, &c., - - - - - - - - - - - - - 169

### EDITORIAL.

Sydenham Society, - - - - - - - - - - - 171

Medicinal Extracts, - - - - - - - - - - - 173

Delegates to the Convention for Revising the National Pharmacopœia, 173

Cholera, - - - - - - - - - - - - - - 174

American Medical Association.—Third Annual Meeting, - - 174

### RECORD OF MEDICAL SCIENCE.

### ANATOMY AND PHYSIOLOGY.

An Account of a Chemico-Gelatinous Injection, with the method of employing it in Anatomical Preparations. By Henry Goadby, Esq., F. L. S., late Dissector of Minute Anatomy to the Royal College of Surgeons of England, - - - - - - - 176

On the Decomposition of the Bile of the Ox. By Dr. Buchner, Jr., - 181

On the Nature of the Gastric Juice, and the Changes it effects on Nitrogenized and Unnitrogenized Food. By H. Bence Jones, M. D., A. M., Cantab., F. R. S., Physician to St. George's Hospital, - - - - - - - - - - 182

MATERIA MEDICA AND THERAPEUTICS.

M. Recamier's Aloetico-Febrifugic Elixir, - - - - - 185

On the Treatment of Psoriasis and Lepra Vulgaris. By M. Emery, 186

On Aconite in Dysentery. By M. Marbot, - - - - - 187

SURGERY.

Case of Elongation of the Under Jaw, and Distortion of the Face and Neck, caused by a burn, successfully treated. By Dr. S. P. Hullihen, of Wheeling, Va., - - - - - - 188

OBSTETRICS.

Prolapsus Uteri during Parturition. By Dr. Harting, - - - 194

On Plugging the Vagina with Vulcanized Caoutchouc. By M. Diday, 194

On Diminution of the size of the Child by Regimen and Venesection. By M. Depaul, - - - - - - - - - 195

Vagitus Uterinus, - - - - - - - - - - - 197

